# Post-traumatic hypoxia exacerbates neurological deficit, neuroinflammation and cerebral metabolism in rats with diffuse traumatic brain injury

**DOI:** 10.1186/1742-2094-8-147

**Published:** 2011-10-28

**Authors:** Edwin B Yan, Sarah C Hellewell, Bo-Michael Bellander, Doreen A Agyapomaa, M Cristina Morganti-Kossmann

**Affiliations:** 1 National Trauma Research Institute, The Alfred Hospital, 89 Commercial Road, Melbourne 3004, Australia; 2 Department of Surgery, Monash University, 89 Commercial Road, Melbourne 3004, Australia; 3 Department of Medicine, Monash University, 89 Commercial Road, Melbourne 3004, Australia; 4 Department of Clinical Neuroscience, Section for Neurosurgery, Karolinska University Hospital, Karolinskavägen, Solna, Stockholm 171 76, Sweden

**Keywords:** Traumatic brain injury, traumatic axonal injury, hypoxia, neurological deficit, cytokine, brain edema, ventricle, metabolism

## Abstract

**Background:**

The combination of diffuse brain injury with a hypoxic insult is associated with poor outcomes in patients with traumatic brain injury. In this study, we investigated the impact of post-traumatic hypoxia in amplifying secondary brain damage using a rat model of diffuse traumatic axonal injury (TAI). Rats were examined for behavioral and sensorimotor deficits, increased brain production of inflammatory cytokines, formation of cerebral edema, changes in brain metabolism and enlargement of the lateral ventricles.

**Methods:**

Adult male Sprague-Dawley rats were subjected to diffuse TAI using the Marmarou impact-acceleration model. Subsequently, rats underwent a 30-minute period of hypoxic (12% O_2_/88% N_2_) or normoxic (22% O_2_/78% N_2_) ventilation. Hypoxia-only and sham surgery groups (without TAI) received 30 minutes of hypoxic or normoxic ventilation, respectively. The parameters examined included: 1) behavioural and sensorimotor deficit using the Rotarod, beam walk and adhesive tape removal tests, and voluntary open field exploration behavior; 2) formation of cerebral edema by the wet-dry tissue weight ratio method; 3) enlargement of the lateral ventricles; 4) production of inflammatory cytokines; and 5) real-time brain metabolite changes as assessed by microdialysis technique.

**Results:**

TAI rats showed significant deficits in sensorimotor function, and developed substantial edema and ventricular enlargement when compared to shams. The additional hypoxic insult significantly exacerbated behavioural deficits and the cortical production of the pro-inflammatory cytokines IL-6, IL-1β and TNF but did not further enhance edema. TAI and particularly TAI+Hx rats experienced a substantial metabolic depression with respect to glucose, lactate, and glutamate levels.

**Conclusion:**

Altogether, aggravated behavioural deficits observed in rats with diffuse TAI combined with hypoxia may be induced by enhanced neuroinflammation, and a prolonged period of metabolic dysfunction.

## Background

Traumatic brain injury (TBI) remains a major health burden in both developed and developing countries. TBI consists of two temporal pathological phases spanning the initial traumatic impact and a multitude of secondary cascades, resulting in progressive tissue degeneration and neurological impairment [1-3]. The pathological consequences of TBI can be variable and largely depend on the presentation of injury as either focal or diffuse, or a combination of both. Diffuse brain injury may result from rotational forces and/or acceleration/deceleration of the head during a traumatic impact, often leading to diffuse axonal injury. Although difficult to diagnose due to the absence of lesions or overt pathology [4,5], diffuse axonal injury is a common presentation, accounting for up to 70% of all TBI cases [6]. The pathology of diffuse axonal injury develops over a delayed time course, and is frequently aggravated by the occurrence of subsequent insults, which are known to worsen morbidity and mortality in TBI patients [7]. Epidemiological studies have revealed that up to 44% of severe head trauma patients experience brain hypoxia, which has been associated with adverse neurological outcomes [8-13]. Hypoxia can be initiated by TBI-induced cerebral hypoperfusion, apnoea and hypoventilation mostly related to brainstem injury [14-16]. In addition, systemic hypoxia can be caused by extracranial injuries often co-existing with head trauma such as obstructed airways, lung puncture and excessive blood loss [9,17]. Despite these clinical observations, the exact mechanisms leading to the exacerbation of brain damage concomitant to posttraumatic hypoxia remain to be elucidated.

One putative sequel of TBI in contributing to secondary tissue damage is the activation of cellular and humoral neuroinflammation. This response is characterised by the accumulation of inflammatory cells in the injured area, as well as the release of pro- and anti-inflammatory cytokines, which may either promote the repair of injured tissue, or cause additional damage [18]. The activation of inflammatory cascades in human and rodent TBI have previously been reported [19-21]. In severe TBI patients, ourselves and others have demonstrated a robust longitudinal increase of multiple cytokines and chemokines in cerebrospinal fluid (CSF) [22-27]. More recently, these findings have been corroborated with the upregulation of TNF, IL-1β, IL-6, IFN-γ protein and gene expression in post-mortem human brain tissue after acute TBI [28]. Animal models of brain hypoxia or trauma can independently activate acute expression of cytokines IL-1β, IL-6 and TNF [29-31]. Furthermore, in models of focal TBI, additional post-traumatic hypoxia was shown to worsen brain tissue damage [32-34], cerebral edema [35], and exacerbate sensorimotor, behavioural and cognitive impairment [32,34,36-38]. The detrimental role of neuroinflammation can be elicited by its ability to induce the production of excitotoxic substances including reactive oxygen and nitrogen radicals [39-41] contributing to the development of brain edema [42,43], blood brain barrier (BBB) disruption [44,45], and apoptotic cell death [43,46-49]. However, almost all the studies on post-TBI hypoxia used focal brain injury models, while epidemiological data on large patient populations reported that the majority of TBI patients present with diffuse brain injury leading to worse neurological outcome especially if associated with hypoxia [6]. The few studies by us and others examining the effect of post-traumatic hypoxia after diffuse traumatic axonal injury (TAI; the experimental counterpart of human diffuse axonal injury) have demonstrated enhanced neurological deficits [34,38], exacerbated edema and cerebral blood flow, and diminished vascular reactivity [50-54]. In a recent study using the Marmarou rat model of diffuse TAI with additional post-trauma systemic hypoxia, we demonstrated a greater axonal damage in the corpus callosum and brainstem co-localising with a robust macrophage infiltration and enhanced astrogliosis, when compared with TAI animals without hypoxia [54-56]. Therefore, using this model of TAI, we aimed to further investigate whether post-traumatic hypoxia also aggravates behavioural and sensorimotor function, cerebral edema, enlargement of lateral ventricles, production of inflammatory cytokines in the brain, and impairment in cerebral energy metabolism.

## Methods

### Induction of trauma

Animal experiments were conducted in accordance with the Code of Practice for the Care and Use of Animals for Scientific Purposes (National Health and Medical Research Council, Australia), and received approval from the institutional Animal Ethics Committee. Adult male Sprague-Dawley rats were housed under a 12-hour light/dark cycle with food and water *ad libitum*. Rats aged 12-16 weeks and weighing 350-375 g on the day of surgery were subjected to TAI (n = 27), TAI followed by a 30-min systemic hypoxia (TAI+Hx; n = 27), hypoxia only (n = 27) or sham surgery (n = 27). Briefly, rats were anaesthetized in a mixture of 5% isoflurane in 22% O_2_/78% N_2_, intubated, and mechanically ventilated with a maintenance dose of 2-3% isoflurane in 22% O_2_/78% N_2_. A steel disc (10 mm in diameter and 3 mm thickness) was adhered to the skull between bregma and lambda suture lines using dental acrylic. Animals were briefly disconnected from the ventilator and moved onto a foam mattress (Type E polyurethane foam, Foam2Size, VA, USA) underneath a trauma device where a weight of 450 g was allowed to fall freely though a vertical tube from 2 m. Following the impact, animals were reconnected to the ventilator, and ventilated continuously for a further 30 min using an appropriate concentration of isoflurane (0.5-1%) in either hypoxic (12% O_2_/88% N_2_) or normoxic (22% O_2_/78% N_2_) gas mixture. Consistent with the literature [32,36] we have previously demonstrated that such systemic hypoxic conditions result in an sO_2 _of 47 ± 4.3% and pO_2 _of 48.5 ± 3.8 mmHg, and cause a significant hypotensive episode, with mean arterial blood pressure (MABP) dropping to 69.5 ± 29.5 midway through the insult (i.e. 15 min). The reduction of sO_2_, pO_2_, and MABP returned to sham values by 15 min following the conclusion of the hypoxic period [55]. Consistent with the original description of this model by Foda et al. (1994) [40], the intubation and ventilation of rats after injury resulted in a mortality rate of ~10% which was confirmed in our study. When the two insults were combined, there was no significant increase in mortality. Hypoxia-only and sham operated animals were surgically prepared as described for TAI rats with the exception of the traumatic impact, and ventilated with hypoxic or normoxic gas, respectively. Rats were housed in individual cages after surgery and placed on heat pads (37°C) for 24 h to maintain normal body temperature during the recovery period.

### Microdialysis probe implantation

Following trauma, 5 rats from each of TAI, TAI+Hx, hypoxia-only and sham groups were inserted with microdialysis probes into the brain for measuring real-time metabolite changes. If the microdialysis probe was implanted soon after the completion of TAI, high severity of the injury together with the ongoing anesthesia would result in a higher mortality rate. Therefore, we allowed the animals to recover for a period of 4 h before implantation of the microdialysis probe. Rats were then anesthetized by isoflurane, intubated and mechanically ventilated as described above. The head of the animal was immobilized on a stereotactic frame with nose and ear bars (David Kopf Instruments, California, USA). The scalp was opened at the existing suture line, and a 1-mm burr hole was drilled into the skull using a small handheld drill at the coordinates of -4.52 mm to bregma and -2 mm lateral to the midline on left hemisphere. Care was taken not to damage the dura mater. Two shallow holes were drilled posterior and anterior to the burr hole, and screws were inserted to provide anchor points for the microdialysis probe implantation. A guide cannula for CMA12 microdialysis probe was adjusted to 3 mm in length, inserted into the brain and secured in place by using dental cement (Dentsply, PA, USA) to cover both the guide cannula and the anchor screws. Once the dental cement solidified, the microdialysis probe (CMA12, 100 kDa cutoff, CMA Microdialysis, Solna, Sweden) was inserted into the guide tube to a suitable length allowing the semi-permeable membrane exposure outside of the guide tube for direct contact with the brain tissue. The microdialysis probe was immobilized by applying additional dental cement over the probe and guide cannula. At surgery completion, animals were allowed to recover in a microdialysis experimental system (CAM 120, CMA Microdialysis) which consists of a balanced arm with dual channel swivel allowing free movement of the animal and continuous collection of microdialysis samples. The microdialysis probe was perfused at 1 μl/min using artificial cerebrospinal fluid (aCSF, CMA Microdialysis). The effluent was collected as accumulative sample over 3 h (i.e. 180 μl/sample) using an automated refrigerated microdialysis fraction collector (Harvard Apparatus, MA, USA). Samples were transferred to -80°C freezer every 12 h and stored until analysis. At the end of the experimental period, animals were killed and brains were perfusion fixed to identify the location of the microdialysis probe in the cortex. Only the animals with the probe tip in the designated location were included for analysis.

### Assessment of sensorimotor functions

Rats were treated in each group as described above and used for assessment of sensorimotor deficit by the Rotarod test, beam balancing and walking test, and adhesive tape removal from forepaws test (n = 10 per group). Animals were trained for these tasks every second day starting 1 week before surgery. These sensorimotor tests were performed daily after TAI for a week, then on every second day until 14 days. The Rotarod allows assessment of movement coordination and function including motor, sensory and balancing skills. Rats were placed on a rotating cylinder made of 18 rods (1 mm diameter) (Ratek, VIC, Australia). The rotational speed of the device was increased in increments of 3 rpm/5 sec, from 0 to 30 revolutions per minute (rpm). The maximal speed at which the rat was unable to match and failed to stay on the device was recorded. Body balancing and walking was assessed using a beam-walking test, in which rats were placed in the middle of a 2-meter long, 2-cm wide beam suspended 60 cm above the ground between 2 platforms. Rats were scored as: [1] normal walking for at least 1 meter on the beam; [2] crawling on the beam for at least 1 m with abdomen touching the beam; [3] ability to stay on the beam but failure to move; and [4] inability to balance on the beam. Sensory and fine motor function was assessed by the ability to remove adhesive tapes (5 × 10 mm; masking tape, Norton Tapes, NSW, Australia) placed on the back of each forepaw. The number of tapes removed (0, 1 or 2) and the latency for each tape removal were recorded within a 2-minute period.

### Open field test

This test evaluates the animal's normal exploratory behavior. Rats were placed in an empty arena (70 × 70 × 60 cm, W×L×H) within an enclosed environment and low lighting. The movement of the rats was recorded for 5 min by a camera, and the distance walked was calculated using a custom made automated movement-tracking program (Dr Alan Zhang, Department of Electrical Engineering, The University of Melbourne).

### Brain edema measurement

Rats with TAI, TAI+Hx, hypoxia or sham surgery were generated for assessment of brain edema. The wet-dry weight method was used for determining the water content of the brain at 2, 24, 48, 72, and 96 h after treatment (n = 6 per timepoint per group). Briefly, the left hemisphere was separated from the rest of brain tissue, weighed on a precision microbalance (Ohaus Adventurer Analytical Balance Bradford, MA, USA), and dried in an oven at 100°C for 24 h. The dry tissue was weighed again, and cortical water content was calculated as ([wet tissue weight - dry tissue weight]/wet tissue weight) × 100.

### Measurement of ventricle size

A cohort of rats for each experimental group was treated as described above and killed at 1 or 7 days after injury (n = 6 per group per timepoint). Brains were perfusion fixed using 4% paraformaldehyde and embedded in paraffin wax. Brain tissue blocks were cut into 10 μm sections at the level of +1 mm relative to the bregma and collected onto glass slides. Sections were dewaxed, rehydrated, stained using hemotoxylin and eosin, and visualized under a light microscope (Olympus BX50). Multiple photographs were taken under 200× magnification to cover the entire sections. Image analysis software (ImageJ, NIH, USA) was used to align images taken from the same brain section to reconstruct a full section view. The whole brain area and the area of the ventricle were measured using ImageJ, with the area of the ventricle expressed as the percentage of total brain area.

### Cytokine measurements

The right hemisphere from each animal of edema study was dissected, the cortex isolated, and stored at -80°C until use. The cortex was homogenised in an extraction solution containing Tris-HCl (50 mmol/L, pH 7.2), NaCl (150 mmol/L), 1% Triton X-100, and 1 μg/mL protease inhibitor cocktail (Complete tablet; Roche Diagnostics, Basel, Switzerland) and agitated for 90 min at 4°C. Tissue homogenates were centrifuged at 2000 rpm for 10 min, and the supernatants stored at -80°C until use. The concentration of 6 cytokines (IL-1β, IL-2, IL-4, IL-6, IL-10, TNF) in the brain cortex homogenates was determined by multiplex assay as previously used in our group [57] (Bio-Rad Laboratories, Hercules, CA, USA). Briefly, colored beads conjugated with cytokine antibodies were loaded into wells of 96-well filter plate. Following washing, the standards, quality controls and samples were added into the wells and incubated overnight at 4°C on a shaking platform. The wells were washed by filtration, and subsequently a solution with a mixture of biotinylated antibodies against each cytokine was added and incubated for 1 h at room temperature. Following the removal of excessive detection antibodies, streptavidin-phycoerythrin was added. Cytokine concentration was measured using multiplex assay reader (Bio-Rad Laboratories) and calculated against the standard curve. Total protein concentration was determined in each sample using the Bradford Assay (Bio-Rad Laboratories).

### Analysis of microdialysis samples

The microdialysis samples (180 μl/sample, n = 5 per group) were freeze dried and suspended in small volume of ddH_2_O to increase the concentration of solutes. The samples were then analysed for glucose, lactate and glutamate using conventional enzymatic techniques performed in the ISCUS Analyser (CMA Microdialysis). Due to a substantial time delay between sample collection and analysis, pyruvate was not measured as it is known to be unstable after storage time of more than 3 months (CMA Microdialysis). The concentrations of glucose, lactate and glutamate in each sample were calculated to the original concentration according to the sample volume before and after the freeze-drying procedure.

### Data analysis

Sensorimotor function assessment, cytokine concentration, brain metabolites and brain edema results were analysed using two-way repeated measures ANOVA. The open field test and ventricular size measurement were analysed by 1-way ANOVA. Data were presented as mean ± standard error of the mean. Data were considered as significant where p < 0.05.

## Results

### Neurological outcome

The impact of post-TAI hypoxia on neurological dysfunction was explored using a number of sensorimotor tests over a period of 2 weeks in TAI, TAI+Hx, hypoxia alone and sham operated animal groups.

### TAI+Hx rats show greater deficits on the Rotarod compared to TAI

**Figure 1:**
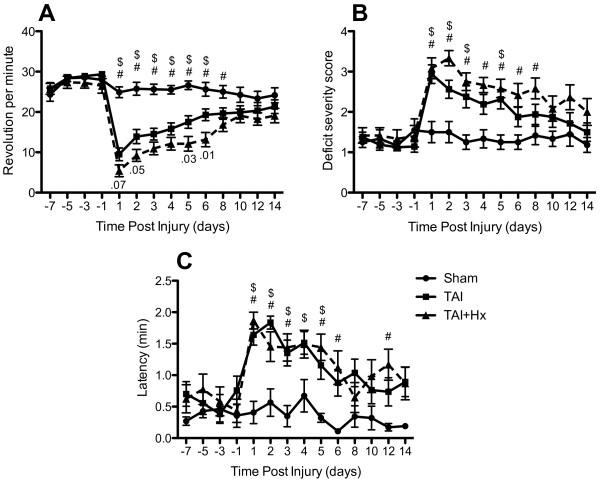
**Sensorimotor function is aggravated following traumatic axonal injury combined with 30 min hypoxia**. Graphics show changes observed over 14 days for the 3 tests employed: (A) Rotarod, (B) beam walking and (C) adhesive tape removal from the front paws. Animals were trained for these tasks for 7 days before trauma, and then tested daily for 6 days after surgery and on every second day until 14 days. $ indicates significant decrease in motor function on the Rotarod, and increase in beam walking deficit score and latency of adhesive tape removal between TAI and sham animals, while # indicates significant difference in these tests between TAI+Hx and sham animals. Numbers in (A) represent the p-values indicating significant differences between TAI and TAI+Hx at days 2, 5 and 6; and close to significant at day 1. The results indicate that TAI+Hx rats require a longer period for neurological recovery towards sham levels, with significant differences between TAI and TAI+Hx rats in the Rotarod test during the first 6 days post-injury. Although a similar deficit on the tape removal test was observed in TAI and TAI+Hx groups versus sham in the first 5 days, TAI+Hx rats exhibited prolonged impairment over sham controls at 6 and 12 days. Data shown as mean ± SEM, n = 10 per group per time point. Data was analysed by 2-way ANOVA repeated measures with Bonferroni *post hoc *test, with a p-value of < 0.05 considered significant.

The Rotarod test involves examining complex body movement and coordination, which showed severe impairment in rats following TAI and TAI+Hx when compared with shams. The maximal speed TAI rats were able to maintain on the Rotarod was significantly decreased at day 1 post-TAI (9.5 ± 1.6 rpm) as compared with shams (24.9 ± 1.3 rpm) (p < 0.05). Over time TAI rats showed a gradual improvement in motor function, however the maximal speed recorded on the Rotarod between day 2 and 6 post-injury (13.9 ± 1.8 and 19.3 ± 1.4 rpm, respectively) remained significantly lower than sham control rats (average 25.83 ± 0.59 rpm) (Figure 1A). Although the motor function in TAI rats improved steadily, from 6 days onwards they failed to recover further, showing a plateau speed on Rotarod until 14 days. When compared to TAI-only rats, the TAI+Hx group had substantially greater motor deficits on the Rotarod, as indicated by a significant lower maximal walking speed at day 2 (9.2 ± 1.5 vs 13.9 ± 1.8 rpm), day 5 (12.1 ± 1.8 vs 17.5 ± 1.5 rpm) and day 6 (13.2 ± 1.8 vs 19.3 ± 1.4 rpm) after injury (p < 0.05) (Figure 1A). These TAI+Hx rats also performed significantly worse on the Rotarod as compared to sham at 8 days (17.13 ± 1.81 vs 25 ± 1.55 rpm), demonstrating that this deficit was prolonged as well as enhanced in rats subjected to the combination of TAI and Hx.

### Ability to balance and walk on a narrow beam is impaired after TAI and TAI+Hx

The beam walk is a sensitive test to determine the ability of injured rats to balance and walk on a narrow beam. TAI and TAI+Hx induced severe impairment on the beam walking test, whereby rats of both groups were unable to balance or stay on the beam at 1 day post-injury (Figure 1B). The deficit scores of beam walking were significantly elevated in both TAI and TAI+Hx groups, particularly during the first 5 days. When compared to sham, TAI only rats displayed a motor impairment which resolved after 5 days. On the contrary, TAI+Hx rats had a significantly greater deficit in walking and balancing compared to sham controls which persisted up to 8 days after injury. Overall, there was no significant difference in beam walking test between TAI and TAI+Hx groups, with both groups returning to sham function by 10 days post TAI or TAI+Hx.

### TAI+Hx rats have prolonged deficits in the adhesive tape removal task

Both TAI and TAI+Hx rats took significantly longer to sense, and subsequently remove the adhesive tapes adhered on the back of forepaws (Figure 1C). In TAI rats significant differences to sham function were detected until day 5. The additional hypoxic insult post-TAI caused further significant differences in latency of adhesive tape removal on days 6 and 12 as compared with TAI-only rats (latency 1.12 ± 0.27 vs 0.88 ± 0.21 min (day 6), 1.23 ± 0.26 vs 0.74 ± 0.22 min (day 12)).

Sham and hypoxia alone (not shown) rats did not change their performance on the Rotarod, beam walking and adhesive tape removal tests over the duration of testing period.

### Voluntary walking in an open field is compromised after TAI+Hx

**Figure 2:**
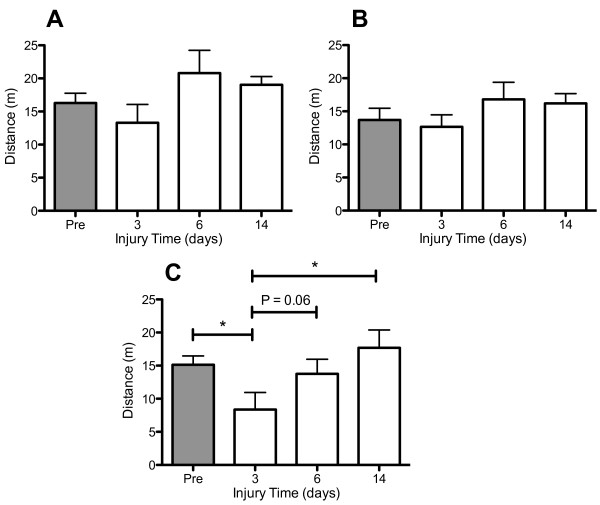
**Spontaneous movement is only reduced after traumatic axonal injury with additional hypoxia**. Distance travelled (metres) was measured for 5 min as indicative of voluntary mobility in a novel open space. Diagrams depict: (A) Sham, (B) TAI, and (C) TAI+Hx. * indicates significant differences between testing at the pre-injury (Pre) or post-injury at days 3, 6 and 14. Distance travelled is shown as mean ± SEM, n = 10 per group per time point. Note the significant reduction in walking distance in TAI+Hx rats at 3 and 6 days as compared to TAI and sham rats. Data was analysed by 1-way ANOVA with Bonferroni *post hoc *test, with a p-value of < 0.05 considered significant.

The ability of voluntary movement was determined by calculating the distance traveled during the first 5 min after the rats were placed in a testing chamber. In the sham group, rats traveled between 12.3 ± 2.8 m and 20.8 ± 3.4 m either before sham operation or at days 3, 6 and 14 days post-surgery (Figure 2A). Hypoxia alone did not alter the distance traveled, which was maintained at sham levels with no differences before or after the insult (data not shown). In comparison to the above sensorimotor function testing, TAI alone did not reduce the voluntary walking distance at 3, 6 or 14 days post-TAI over the pre-TAI levels (Figure 2B). However, an additional hypoxic insult after TAI significantly decreased the mobility of rats to 55.2% of the pre-TAI+Hx level at day 3 post-injury (8.4 ± 2.6 m vs 15.1 ± 1.3 m, respectively; p < 0.05) (Figure 2C). By day 6, the distance of voluntary movement in TAI+Hx rats was slightly increased (13.8 ± 2.2 m; p = 0.06) and was fully restored to pre-TAI+Hx level at day 14 (17.7 ± 2.8 m) after injury.

### Brain water content is elevated after TAI and TAI+Hx

**Figure 3:**
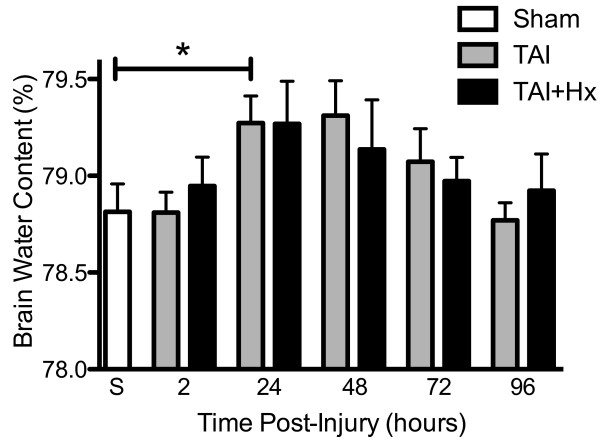
**Increase in brain edema does not differ in traumatic axonal injury rats with or without hypoxia**. Brain water content was determined at 2, 24, 48, 72 and 96 h post-injury, and calculated as percentage of dry and wet ratio in the brain of sham (S), TAI alone, and TAI with hypoxia (TAI+Hx) animals. * indicates significant difference between groups. Both TAI and TAI+Hx showed similar increases in brain water content, and no differences were found between these groups. Data shown as mean ± SEM, n = 6 per group per time point. Data was analysed by 1-way ANOVA with Bonferroni *post hoc *test, with a p-value < 0.05 considered significant.

Cerebral edema is a common pathophysiological consequence in this model of TAI [35,58,59]. Using the wet-dry ratio method, we showed that brain water contents in hypoxia-only and sham animals were within the normal ranges reported in the literature [60] and remained unchanged over time (not shown). In contrast, whilst the brain water content of TAI and TAI+Hx rats was similar to shams at 2 h post injury, by 24 h, it increased significantly in TAI rats when compared with sham (79.27 ± 0.14% vs 78.81 ± 0.14%, respectively; p < 0.05; Figure 3) and increased to near significance between TAI+Hx and sham (79.27 ± 0.22% vs 78.81 ± 0.14%, respectively; p = 0.1147). The brain water content remained elevated in both trauma groups for 48 h after injury, and then decreased to sham levels by 72 h. Overall, brain water content was similar in TAI and TAI+Hx groups at all time points examined.

### The lateral ventricles are enlarged after TAI and TAI+Hx

**Figure 4:**
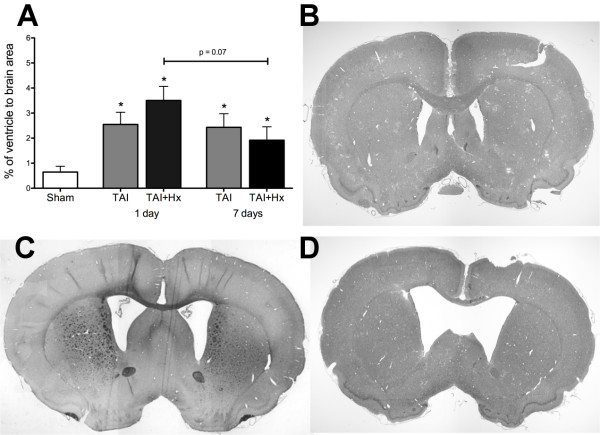
**Ventricular enlargement**. Enlargement of the lateral ventricles following TAI and TAI with hypoxia (TAI+Hx) was quantified by expressing the ventricle size as percentage of the entire brain section (A), at coronal plane of +1.0 mm to bregma in accordance with rat atlas by Paxinos and Watson [61]. Coronal sections of (B) sham, (C) TAI alone and (D) TAI+Hx taken at +1 mm to bregma at 1 day after injury. * indicates significant differences to sham group. Data shown as mean ± SEM, n = 6 per group per time point. Data was analysed by 1-way ANOVA with Bonferroni *post hoc *test, with a p-value of < 0.05 considered significant.

We measured the changes in lateral ventricle at +1.0 mm to bregma in concurrence with Paxinos and Watson rat brain atlas [61]. Ventricular size was unchanged at all timepoints in animals that underwent sham surgery or hypoxia alone (data not shown). The ventricles of TAI animals were significantly enlarged 1 day post-injury when compared to sham (2.55 ± 0.49% vs 0.65 ± 0.23%, p < 0.01; Figure 4A, B, C). Post-TAI hypoxia resulted in a further, non significant increase in the size of the ventricles at 1 day (3.50 ± 0.57%; Figure 4D) when compared with TAI only rats (2.55 ± 0.49%). This size was 5.4-fold larger than sham (3.50 ± 0.57% vs 0.65 ± 0.23%; p < 0.001) (Figure 4A). By 7 days, although the ventricular size was reduced as compared to day 1, they were still larger than sham control rats being 2.43 ± 0.54% in TAI and 2.04 ± 0.45% in TAI+Hx animals.

### The production of cytokines is enhanced following TAI+Hx

**Figure 5:**
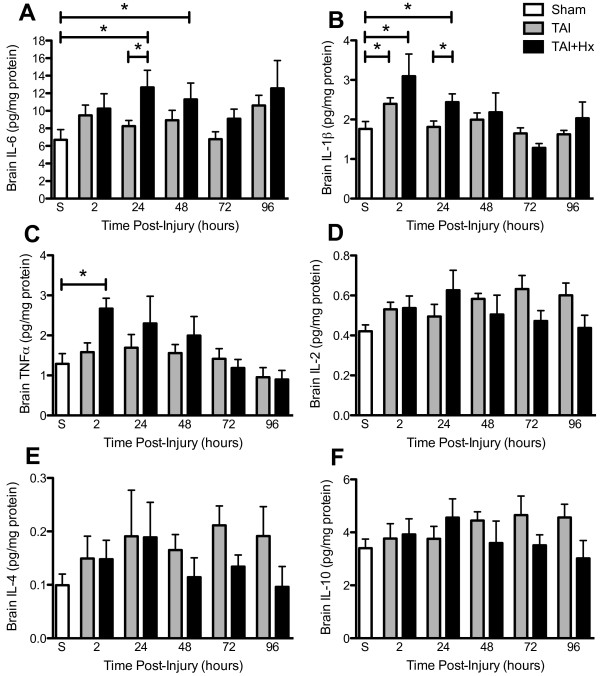
**Cytokines IL-6, IL-1β and TNF are increased in rats after traumatic axonal injury with additional hypoxia**. The concentration (pg/mg protein) of cytokines (A) IL-6, (B) IL-1β, (C) TNF, (D) IL-2, (E) IL-4 and (F) IL-10 was measured in cortical homogenates of sham (S), TAI alone, and TAI with hypoxia (TAI+Hx) animals by multiplex assay over 4 days. * indicates significant differences between groups. Note the significant increases of IL-6 and IL-1β in TAI+Hx vs TAI rats. TNF did not increase after TAI alone, and was only evident at 2 h in TAI+Hx rats. Data shown as mean ± SEM, n = 6 per group per time point. Data was analysed by 1-way ANOVA with Bonferroni *post hoc *test, with a p-value of < 0.05 considered significant.

The neuroinflammatory response was determined by measuring changes in cytokine production in the homogenised cortex over 4 days (Figure 5). In these experiments six cytokines were measured: IL-6, IL-1β, TNF, IL-2, IL-4 and IL-10. However, relevant differences were only detected in three of them, IL-6, IL-1β and TNF. For the other cytokines including the pro-inflammatory IL-2 and anti-inflammatory IL-4 and IL-10, no changes were detected in either the TAI or TAI+Hx groups, with values remaining comparable to those of sham animals over time (Figure 5D-F). Hypoxia alone did not induce any changes in brain cytokine concentration at any time points (data not shown).

### IL-6

In comparison to the cytokines measured in these experiments, IL-6 presented the highest concentration in the injured cortex. By 2-way ANOVA, the overall increase of IL-6 (all time points within the group analysed together) was significantly more elevated in TAI+Hx brains when compared to either the sham or TAI groups (p < 0.05, Figure 5A), while no changes were observed between sham and TAI animals. Using *post hoc *analysis, we demonstrated that hypoxia following TAI significantly increased the concentration of IL-6 in the brain at 24 h (12.67 ± 1.95 pg/mg protein) and 48 h (11.30 ± 1.86 pg/mg protein) when compared with sham animals (6.71 ± 1.17 pg/mg protein, p < 0.05). In addition, TAI+Hx rats had significantly higher IL-6 levels than TAI rats at 24 h post-injury (12.67 ± 1.95 pg/mg protein vs 8.26 ± 0.65 pg/mg protein; p < 0.05).

### IL-1β

In contrast to IL-6, the elevation of IL-1β occurred earlier and transiently after TAI (Figure 5B). In the TAI group, a significant increase was observed 2 h post injury (2.40 ± 0.15 pg/mg protein) as compared with sham (1.76 ± 0.68 pg/mg protein; p < 0.05). In the TAI+Hx group, a more striking significant increase was observed at both 2 h (3.10 ± 0.56 pg/mg protein) and 24 h (2.44 ± 0.21 pg/mg protein) as compared with sham (p < 0.05). A significant difference was also found between TAI and TAI+Hx at 24 h post injury (1.81 ± 0.15 pg/mg protein vs 2.44 ± 0.21 pg/mg protein; p < 0.05). The concentration of IL-1β in both injury groups returned to sham levels at 48 h post-injury.

### TNF

No increase in TNF was detected at any timepoint examined in the TAI group. Instead, similarly to IL-1β, the concentration of TNF in the brain of TAI+Hx rats was significantly increased at 2 h when compared with sham controls (2.67 ± 0.26 pg/mg protein vs 1.29 ± 0.26 pg/mg protein; p < 0.05). In TAI+Hx group TNF rapidly returned close to the sham level at 24 h (Figure 5C).

### Changes in metabolism after TAI and TAI+Hx

TBI is known to result in a reduction of oxidative metabolism [62]. We expected post-TAI hypoxia to aggravate the metabolic disarray caused by diffuse axonal injury and employed the microdialysis technique to monitor changes of various metabolites over 4 days. Due to the detection of significant alterations in brain metabolites following the implantation of microdialysis probe in uninjured sham animals as reported by others [63], we chose to discard samples over the first 20 h following probe implantation to reduce the artifact from the needle injury. In this study we were only present data of glucose, lactate and glutamate from the microdialysates, since pyruvate is known to become unstable after prolonged storage time (CMA Microdialysis).

### Depression of glucose metabolism is prolonged after TAI+Hx

**Figure 6:**
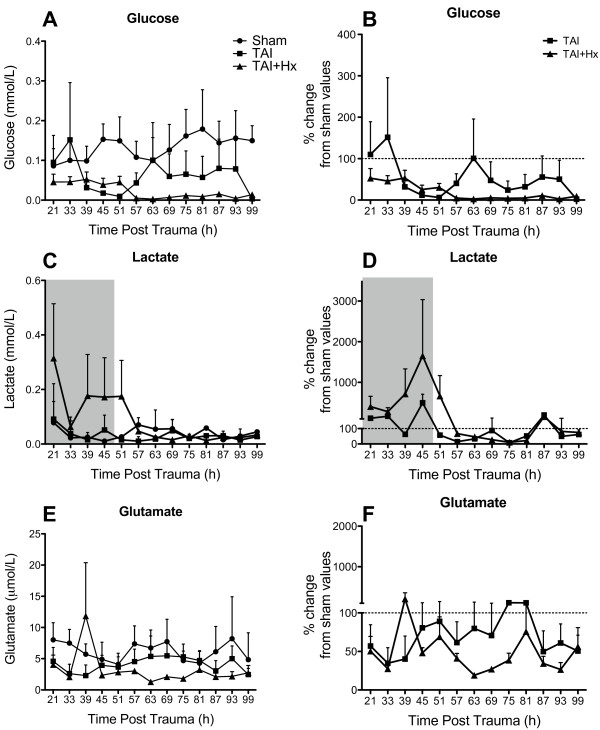
**Metabolic alterations are exacerbated in rats exposed to traumatic axonal injury with additional hypoxia**. Cerebral microdialysis samples were analysed between 21 h and 99 h after sham surgery, TAI and TAI with 30 min hypoxia (TAI+Hx). Data are expressed as both raw values and percentage changes from sham values for glucose (A, raw values; B, % change from sham levels), lactate (C, raw values; D, % change from sham levels) and glutamate (E, raw values; F, % change from sham). Shaded area in (C) and (D) represents the peak period of edema, which correlated with maximal lactate production. Overall a significant hypoglycaemic response was observed in both the TAI and TAI+Hx groups compared to shams (2-way repeated measures ANOVA, p < 0.05). Data shown as mean ± SEM, n = 5 per group per time point. Data was analysed by 2-way ANOVA repeated measures, with a p-value of < 0.05 considered significant.

Overall a significant hypoglycemia was observed in both TAI and TAI+Hx groups when compared with sham (p < 0.0001, Figure 6A). At 21 h post injury the concentration of glucose in TAI rats was similar to sham (0.09 ± 0.06 mmol/L vs 0.09 ± 0.04 mmol/L) and remained similar until 33 h, after which time a substantial decrease was observed, with glucose levels dropping to 30% of sham values (0.03 ± 0.02 mmol/L vs 0.09 ± 0.04 mmol/L) (Figure 6A &6B). Glucose levels remained low until 51 h post-injury, when values gradually increased toward to sham levels before they dropped again below sham levels from 69 h until the end of experiment. In TAI+Hx rats, glucose levels in the microdialysate were approximately 50% lower than the levels of sham or TAI rats at 21 h (0.04 ± 0.02 mmol/L vs 0.09 ± 0.06 mmol/L and 0.09 ± 0.04 mmol/L, respectively) (Figure 6A &6B), with these low values subsisting until 51 h. While the TAI rats showed some elevation in glucose levels after 51 h, TAI+Hx rats had the opposite pattern, with values further decreasing to less than 10% of those observed in sham, (0.005 ± 0.002 mmol/L), and remaining under 10% of sham values for the study period.

### Lactate is elevated after TAI+Hx and coincides with the peak period of edema

Lactate levels in the microdialysates of sham-treated animals remained low for the duration of the study. Lactate measurements for TAI animals were similar to sham levels from 21 h until 51 h, when a substantial decrease was observed to values less than 60% of sham (Figure 6C &6D). Values in TAI rats remained lower than sham until 87 h, when lactate levels recovered to sham-level were observations. Although not statistically significant, TAI+Hx rats had lactate levels which were 400% higher at 21 h when compared to sham or TAI (0.31 ± 0.2 mmol/L vs 0.08 ± 0.03 mmol/L and 0.09 ± 0.05 mmol/L, respectively; Figure 6C &6D). The lactate levels in TAI+Hx rats increased until 51 h correlating with the peak period of edema observed in this study (shaded area, Figure 6C &6D), then rapidly decreased to 10% of sham levels at 75 h before recovering to sham- and TAI-levels by 87 h (0.05 ± 0.01 mmol/L vs 0.05 ± 0.02 mmol/L and 0.01 ± 0.01 mmol/L, respectively).

### Glutamate level is depressed after TAI and TAI+Hx

Glutamate levels in TAI animals at 21 h were approximately 40% less than those observed in sham animals (4.58 ± 2.21 μmol/L vs 8.02 ± 2.74 μmol/L; Figure 6E &6F), and remained low until 45 h, at which point the microdialysate levels returned to sham levels until 87 h, when another decrease was observed. TAI+Hx rats had glutamate levels of approximately 50% of sham at 21 h, and though not significant, a peak was observed at 39 h to more than 200% of sham values (11.84 ± 8.54 μmol/L vs 5.71 ± 1.64 μmol/L; Figure 6E &6F). From this time onwards, glutamate levels in TAI+Hx rats decreased again to 50% of sham values, and remained at between 30-60% of shams for the remainder of the experimental period.

## Discussion

Cerebral hypoxia, along with hypotension, is one of the most critical factors worsening secondary brain damage after TBI, and particularly following diffuse TBI [6,13]. Despite this clinical relevance, the underlying mechanisms by which hypoxia aggravates neurological outcome following TBI have not been studied adequately.

Using focal or mixed focal-diffuse models, systemic hypoxia following TBI in rats exacerbates neurological deficit [32,37] and increases the lesion size, neuronal death [33,34,37,64] and brain edema, while reducing cerebral blood flow [35,51]. However, the role of post-traumatic hypoxia elicited after diffuse brain injury has rarely been addressed. Therefore, we explored the impact of hypoxia using a model of diffuse TAI [40,65,66] followed by a 30-min hypoxic ventilation. Using this combinatorial insult model, we previously reported enhanced axonal damage and macrophage infiltration within the corpus callosum and the brain stem [55]. Thus, in this follow-up study we further investigated changes in neurological outcome, brain edema, ventricle enlargement, cerebral cytokines, and energy metabolism.

We found that in comparison to TAI alone, an additional hypoxic insult enhanced sensorimotor deficits on the Rotarod, beam walk and tape removal tests, reduced spontaneous exploratory behavior, and delayed recovery. These data closely relate to clinical studies on TBI patients showing that post-traumatic hypoxia worsens neurological outcome and prolongs the recovery period [7,8,67]. The behavioural data in this model of TAI are consistent with similar deficits shown at day 1 in previous studies using diffuse or focal TBI models in combination with hypoxia [32,34,36-38,68]. However, in extension of this early work, our results show that an additional hypoxic insult has a detrimental effect on behaviour, inflammatory and metabolic outcomes for an extended period of time.

Brain swelling is a major contributor for the development of secondary ischemia causing raised ICP and decreased cerebral perfusion pressure [69]. Enlargement of the brain due to edema [70] and/or obstruction of CSF flow [71] is a common event in severe TBI patients and a frequent cause of death. Cytotoxic edema results from excessive accumulation of ion and water within the cell, while vasogenic edema is caused by increased vascular permeability and subsequent fluid extravasation into the parenchyma. Here, we demonstrated that at 2 h after TAI, brain water content was similar to sham animals, but it increased to a peak between 24 and 48 h, and remained elevated until 72 h. Although hypoxia following TAI exacerbated sensorimotor deficit, it did not further increase cerebral edema when compared with TAI only animals, corroborating previous observations using diffuse-weighted imaging [35]. Interestingly, using MRI, others demonstrated that acute brain swelling after TAI (both with and without hypoxia), as early as 60 min post-injury, was associated with increased extracellular fluid and BBB dysfunction, indicative of vasogenic edema [72-75]. This early brain swelling was transient, with values quickly returning to sham levels [53,58,75]. Since the earliest timepoint examined in our study was 2 h, it is likely that we missed this initial peak in edema, as no differences were detected between TAI, TAI+Hx and sham rats later on. However, other studies have also demonstrated that a modest, widespread second edematous response occurs at 24 h after TAI despite the intact BBB, which suggests ongoing cytotoxic edema [58,75]. Our results are consistent with this modest yet significant increase of edema at 24 h, which was maintained until 48 h. It is possible that the peak in brain water content observed at 24 h in both the TAI and TAI+Hx rats (approximately 79.3%) reflects a sort of saturation level, with the brain unable to tolerate any further water accumulation. Other studies also demonstrated peak edema of similar degree after TBI [59,76,77].

An interesting observation was the enlargement of the lateral ventricles after TAI, and even greater following TAI+Hx. Recent clinical neuroimaging studies have shown correlations between ventricular enlargement and long-term neurological impairment [78-80]. The prognostic value of ventricular dilatation had high sensitivity and specificity for the prediction of cognitive outcome [80-83]. In this study, we showed that the lateral ventricles are markedly enlarged at 1 day post-injury after TAI and even larger in TAI+Hx animals, when compared to sham or rats with isolated hypoxia. Although we did not examine the mechanism leading to ventricular enlargement after TAI, imaging studies on TBI patients suggested that white matter degeneration around the lateral ventricle may be a contributing factor [84]. However, since ventricular enlargement in TAI rats was an early and transient effect, it could be most likely attributed to the onset of post-traumatic hydrocephalus, caused by impaired CSF circulation due to edema compressing the aqueduct of sylvius.

Neuroinflammation has been extensively investigated in hypoxia-ischemia and TBI in both humans and animal models [85] and all these studies have reported a robust elevation of cytokines in the central nervous system [19,28,86-89]. More relevant for this study, our preliminary data on severe TBI patients with additional hypoxic insult have shown enhanced and prolonged production of cytokines in the CSF (Yan et al: Neuroinflammation and brain injury markers in TBI patients: Differences in focal and diffuse brain damage, and normoxic or hypoxic status on neurological outcome; manuscript in preparation). Consistently, here we demonstrated exacerbated production of IL-6, IL-1β, and TNF in the brains after TAI with additional hypoxia.

IL-1β is a key mediator of the inflammatory response, which exacerbates neuronal injury and induces BBB dysfunction by stimulating matrix metalloproteinases [90]. IL-1β mRNA is upregulated within minutes after TBI, and increased protein levels are detectable within an hour after TBI [21,91-93]. In this study, IL-1β increased early after TAI alone, peaking at 2 h. Post-TAI hypoxia significantly enhanced IL-1β concentration at 2 h compared to TAI-only rats. In addition, whilst the elevation of IL-1β in TAI-only rats appeared to be transient, in TAI+Hx rats IL-1β was still significantly elevated at 24 h, suggesting that the addition of hypoxia prolongs neuroinflammation.

The neurotoxic effects of IL-1β are synergistically enhanced in the presence of TNF [94], as both share many of the same physiologic effects. However, the role of TNF following TBI is controversial, neuronal toxicity of TNF has been demonstrated with local TNF administration inducing breakdown down of the BBB and increased leukocyte recruitment [95-98]. Clinically, high levels of TNF in the CSF of brain-injured patients correlated with BBB dysfunction [99]. TNF inhibition also reduced cerebral ischemia/reperfusion injury [100], decreased TBI induced neuronal damage [101], and ameliorated BBB dysfunction after closed head injury [102]. However, studies on TNF deficient mice demonstrated an early functional improvement between 24-48 h after TBI, but failed to produce further amelioration at 4 weeks [103]. Taken together, these studies suggest that TNF may be deleterious in the acute phase post-injury, but beneficial for long-term recovery. In accordance with Kamm et al. [93], no changes in TNF levels were detected in rats subjected to TAI alone, whereas the combination of TAI and hypoxia elicited a significant early increase in TNF at 2 h post-injury, lasting up to 72 h post-injury. These early enhancement in the TAI+Hx rats possibly reflects a more severe brain damage in this combined insult model.

Similar to IL-1β and TNF, at 24 h IL-6 was significantly higher in TAI+Hx rats compared to TAI alone. IL-1β is an early mediator inducing the production of IL-6 at both mRNA and protein levels [21]. IL-6 displays pleiotropic functions with both deleterious and beneficial effects in the injured brain [104-106]. Using the mild severity (250 g/2 m) of the Marmarou model, we showed that IL-6 increased in rat CSF within 24 h and IL-6 protein and mRNA was found expressed on neurons [95]. Studies of IL-6 gene-deficient mice have provided more information in regards to the protective function of IL-6, by having a compromised immune response, increased oxidative stress and neurodegeneration [107]. In this study, we demonstrate significantly heightened IL-6 levels in the TAI+Hx rats at 24 h, which remained elevated above TAI levels until 96 h. Altogether, the increased acute production of IL-1β and TNF may be associated with disruption of BBB integrity and consequently formation of cerebral edema, while late elevation of IL-6 may trigger repair mechanisms [24,99].

We also investigated changes in energy metabolism in this combinatorial insult model. Due to the nature of the impact acceleration injury, it is impractical to implant a microdialysis probe prior to injury without compromising the integrity of the trauma. It is also difficult to implant the probe directly after trauma as it resulted in higher mortality rate. Carré and colleagues implanted the probe 2 weeks prior to injury, but without success [108]. We therefore allowed the rats to recover for 4 hours after TAI before implanting the microdialysis probe. In accordance with others [63], our study has shown that in sham rats energy metabolism is altered during the first 24 h following microdialysis probe implantation, therefore we chose to examine only the data from 20 h onwards to reduce the "probe effect".

At 21 h, the glucose values for TAI+Hx rats were substantially lower compared to TAI or sham rats, and dropped to extremely low levels from 57 h onwards. These low levels of cerebral glucose could be the result of low glucose availability and/or hyperglycolysis in the acute post-injury phase. Hyperglycolysis has previously been shown as common early event following neurotrauma both experimentally and in the clinic [109,110]. It is often followed by a prolonged period of metabolic depression beginning as early as 6 h post-injury, remaining for as long as 5 days [111,112], a phenomenon which has also been demonstrated in the present study. Interestingly, rats subjected to TAI experienced only a brief period of glucose depletion between 39 h and 57 h, at which time glucose levels returned to sham values for the remaining duration of monitoring. It is possible that the additional hypoxic insult depleted available glucose stores in the TAI+Hx animals, and thus a prolonged compensatory period of anaerobic respiration occurred to provide essential ATP and generate lactate as by-product. Our experiments have demonstrated that this is a protracted process, lasting for 51 h after TAI. Lactate may be utilized by the brain during periods of increased brain energy requirements in which ATP and glucose stores are exhausted, such as following TBI [113,114]. In a situation of prolonged glucose depletion, high concentrations of lactate and high-level energy usage for neuronal repair or alternative metabolic pathways may further reduce the ATP reserves, with a subsequent mismatch between glucose transport, uptake and ATP production [115,116]. This may explain the further drop in glucose concentrations at 57 h post TAI, in that the restoration of aerobic metabolism decreases lactate concentration but further reduces glucose. Post-traumatic impairment in energy metabolism is a major contributor to cytotoxic edema, and interestingly, the period of elevated lactate in the TAI+Hx rats between 21 h and 57 h overlaps with the peak of increased brain water content. As edema begins to reside, lactate levels in these rats return to sham values. This prolonged period of metabolic crisis also extends to glutamate production, which was depressed below sham levels for TAI, and particularly TAI+Hx rats, for the duration of the monitoring by microdialysis.

## Conclusion

In this study, we reproduced a frequent debilitating condition contributing to poor neurological outcome in humans by using a rat a model of diffuse TAI combined with an hypoxic insult. Consistent with our hypothesis, we demonstrated exacerbation of sensorimotor deficits and delayed neurological recovery in TAI+Hx rats, as well as a significant enlargement of the lateral ventricles after TAI and TAI+Hx. However, no differences were detected in brain edema, which was similarly increased in both TAI and TAI+Hx injury groups. Enhanced neuroinflammation via amplified cerebral production of IL-1β, TNF and IL-6 corroborates our previous findings of exacerbated macrophage/microglial accumulation in regions of axonal pathology in the corpus callosum and brainstem of TAI+Hx animals [55]. Interestingly, while TAI rats had a gradual recovery in glucose levels, metabolic depression was sustained in TAI+Hx rats, showing elevated lactate in microdialysates coinciding with the period of increased brain edema. Overall, the morphological and behavioural changes of this combined model of diffuse TBI and hypoxia has similar characteristic of the reported severe brain damage and poor outcomes in patients with diffuse brain injury and hypoxia.

## List of abbreviations

ATP: adenosine triphosphate; BBB: blood brain barrier; CSF: cerebrospinal fluid; Hx: hypoxia; IFN: interferon; IL: interleukin; MABP: mean arterial blood pressure; MRI: magnetic resonance imaging; pO2: partial pressure of oxygen; rpm: revolutions per minute; sham: sham-operated animals; sO2: oxygen saturation; TAI: traumatic axonal injury; TAI+Hx: traumatic axonal injury with hypoxia; TBI: traumatic brain injury: TNF: tumor necrosis factor.

## Competing interests

The authors declare that they have no competing interests.

## Authors' contributions

EBY designed the study, performed all animal work and microdialysis probe implantation, performed cytokine measurements, drafted the manuscript, and performed statistical analysis. SCH assisted with animal work, performed sensorimotor experiments, carried out the histology and ventricle measurements, performed statistical analysis, and drafted the manuscript. BMB carried out microdialysis sample measurements and assisted with manuscript preparation. DAA assisted with animal work, carried out sensorimotor and open field exploration experiments, and performed edema experiments. CMK conceived of the study and oversaw its design and coordination, and drafted the manuscript. All authors have read and approved the final manuscript.
